# Enhanced Thermal and Storage Stability of Glucose Oxidase via Encapsulation in Chitosan-Coated Alginate and Carboxymethyl Cellulose Gel Particles

**DOI:** 10.3390/foods14040664

**Published:** 2025-02-15

**Authors:** Zhihao Guo, Jian Ren, Chunli Song

**Affiliations:** 1 College of Food and Bioengineering, Qiqihar University, Qiqihar 161006, China; guoxiaobaiy@163.com (Z.G.); renjian@qqhru.edu.cn (J.R.); 2 Engineering Research Center of Plant Food Processing Technology, Ministry of Education, Qiqihar 161006, China

**Keywords:** glucose oxidase, alginate, sodium carboxymethyl cellulose, chitosan coating, enzyme encapsulation, thermal stability, storage stability

## Abstract

Glucose oxidase (GOD) is widely used as an important oxidoreductase in various fields. However, maintaining the vitality and stability of GOD under environmental stress is a challenge. To improve the thermal and storage stability of GOD, this study constructed sodium alginate–carboxymethyl cellulose sodium gel particles (SA/CMC) and chitosan-coated SA/CMC gel particles (CS/SA/CMC) of GOD. The encapsulation efficiency (EE), gel particle structure, stability, and release behavior of GOD were evaluated. The results showed that the thermal stability of GOD encapsulated in SA/CMC and CS/SA/CMC gel particles was improved by approximately 2.8-fold and 4.3-fold compared with the free enzyme at 85 °C, respectively. In addition, CS/SA/CMC gel particles enhanced the enzyme activity retention rate of GOD to over 80% during storage at 4 °C for four weeks. Both SA/CMC and CS/SA/CMC gel particles loaded with GOD had more than 70% of the enzymes released during the simulated gastrointestinal experiment. The results demonstrated that encapsulating GOD in SA/CMC and CS/SA/CMC gel particles could improve its thermal stability and storage stability, which is conducive to further expanding the application of GOD in food, pharmaceutical and feed industries.

## 1. Introduction

Glucose oxidase (GOD) is known to facilitate the conversion of β-D-glucose into D-glucono-δ-lactone, which then naturally breaks down into D-gluconic acid and hydrogen peroxide in the presence of oxygen. GOD is widely utilized in the medical, feed, and food industries [1,2] due to its specificity, high stability, and rapid turnover [3]. However, in practical applications, GOD is often susceptible to various challenges. Industrial methods used in diet production, such as extrusion, pelletizing, heat drying, changes in acidic and alkaline environments, and the presence of metal ions, could compromise the structural integrity of GOD and decrease its functionality [4].

To enhance the stability of GOD, encapsulation is used as an effective strategy to protect GOD against environmental stress [5]. Alginate spheres are commonly utilized for encapsulating enzymes because of their remarkable stability and simple synthesis. Alginate, also known as sodium alginate (SA), is a natural polysaccharide extracted from the cell walls of brown algae and is a linear natural polymer. It has various excellent properties such as high immobilization ability, hydrophilicity, porosity, and thermal stability [6]. The ability to form gels by cross-linking with multivalent ions such as calcium and barium ions makes alginate a popular candidate for encapsulation [7,8]. Although the alginate spheres encapsulation method is easy to synthesize and effective in stabilizing enzymes, it frequently faces challenges such as a low encapsulation efficiency (EE), enzyme leakage, and poor enzyme recycling [9]. The stability of single alginate bead-encapsulated enzymes may not meet the requirements for industrial production. According to previous studies, the EE of enzymes and the stability of gel microspheres can be enhanced by creating composite gels with alginate. This can be achieved by incorporating other polymers, such as carrageenan, gelatin, or gellan, or by applying an additional coating layer, such as chitosan or poly-L-lysine, to the exterior of the alginate beads [10,11].

Reducing water loss during gel formation can effectively increase the EE [12]. Carboxymethyl cellulose (CMC) is a water-soluble cellulose derivative. Due to its ability to form gels, carboxymethyl cellulose in gel products provides excellent water retention properties. This polymer has been used as an additive in the food industry [13,14]. Therefore, SA and CMC as a composite carrier are beneficial to improve the EE of enzymes. At the same time, SA/CMC can partially improve the stability of the encapsulated enzyme based on the core–shell structure and the structural restriction on the enzyme [15,16].

Scholars are also committed to studying chitosan (CS) as a coating and its synergistic application within microcapsules. CS is a natural biopolymer [17], and has the advantages of non-toxicity, biodegradability, and biocompatibility. CS has been widely studied for coating alginate capsules to encapsulate bioactive substances, as its positive charge forms ionic complexes with the negative charge of alginate [10]. CS coating has been shown to provide a blend of gel strength, barrier features, and controlled release properties in microcapsules [18,19,20,21]. Thus, the chitosan coating can improve the thermal stability of the enzyme (neutral protease, 30–80 °C) [22] and protect the enzyme from inactivation by gastric juice [23]. The resulting coating can enhance stability, improve adhesion to mucous membranes, and increase structural density [24,25]. CS coating can improve the protection ability of capsules [26,27,28,29] and microcapsules under gastric conditions [30,31] and enhance the storage stability of bioactive substances (probiotics at 4 °C) [32] and thermal stability (*Bifidobacterium* at 55–65 °C) [33].

To our knowledge, the potential benefits of encapsulating GOD using SA and CMC, as well as CS coating for further encapsulation of GOD in SA/CMC, have not been sufficiently studied or reported. Therefore, the purpose of this study was to encapsulate GOD through the synergy of SA and CMC to improve the EE of the GOD. CS was then added to coat the gel particles to further improve the stability of SA/CMC gel particles. Fourier-transform infrared spectroscopy (FTIR) and scanning electron microscopy (SEM) were used to characterize the gel particle structure. The properties of gel particles were investigated through thermal stability, storage stability, and release characteristics analyses. The results were utilized to develop a novel method for enhancing the stability and activity of GOD, potentially broadening its applications in food, medicine, and feed.

## 2. Materials and Methods

### 2.1. Materials

GOD (40 U/mg) was purchased from Heilongjiang Biotechnology Co., Ltd. (Qiqihar, China). SA was purchased from Fuchen Chemical Reagent Co., Ltd. (Tianjin, China). CMC was purchased from Tianjin Guangfu Fine Chemical Research Institute Co., Ltd. (Tianjin, China). CS (degree of deacetylation ≥ 90.0%) was purchased from Sinopharm Chemical Reagent Co., Ltd. (Beijing, China). Anhydrous calcium chloride was purchased from Tianjin Kermel Chemical Reagent Co., Ltd. (Tianjin, China). D-Anhydrous glucose, horseradish peroxidase (150 U/mg), and *o*-dianisidine were purchased from Shanghai Macklin Biotech Co., Ltd. (Shanghai, China). Pepsin and Trypsin were purchased from Sigma-Aldrich (St. Louis, MO, USA). Other chemical reagents were of analytical grade.

### 2.2. Preparation of SA/CMC Gel Particles

The SA/CMC gel particle loaded with GOD was prepared by the ionic gelation method, as described by Zhao et al. [34], with slight modifications. Using EE as an indicator, single-factor experiments (Figure S1) and orthogonal experiments (Table S2) were conducted on material levels and enzyme concentrations. The optimal preparation process for SA/CMC gel particles was as follows. SA (27 mL, 1.5%, *w*/*v*) and CMC (3 mL, 0.1%, *w*/*v*) were mixed as a gel-forming solution. Subsequently, the GOD solution (1 mL 0.3%, *w*/*v*) was uniformly mixed with SA-CMC aqueous mixture, and bubbles were removed by ultrasound (40 kHz, 5 min) before injecting. The resulting mixed solution was dropped into 100 mL of sterile CaCl_2_ solution (1%, *w*/*v*). A peristaltic pump was used to assist in this process, running at 12 rpm and utilizing a silicone double manifold tube with a 3 mm inner diameter, ending with a 0.45 × 16 RWLB stainless needle with a 0.45 mm inner diameter. The injection height was about 8–10 cm. The obtained gel particles were cured at 4 °C for 30 min and filtered using medical-grade gauze, and the gel particles were washed with sterile water 2 to 3 times after filtration. The water was absorbed with filter paper. SA/CMC gel particles were collected in a centrifuge tube and refrigerated at 4 °C for further use.

### 2.3. Preparation of CS/SA/CMC Gel Particles

In our preliminary experiments, we conducted single-factor experiments (Figure S2) and response surface experiments (Figure S3) using EE as the indicator to study the concentration of CS solution, the pH value of CS solution, and the coating time [5]. The group with the highest EE was selected as follows. The prepared SA/CMC gel particles were placed in 100 mL of CS solution (0.6% *w*/*v*, pH 4.0) and stirred for 30 min at 150 rpm with a magnetic stirrer. Subsequently, the gel particles were filtered with medical-grade gauze and then washed twice with sterile water. The water was absorbed using filter paper. CS/SA/CMC gel particles were obtained.

### 2.4. Calculating GOD Activity

The activity of GOD was estimated by determining the amount of H_2_O_2_ that formed [35]. A standard curve was constructed by the H_2_O_2_ concentration. In the test tube, 2.5 mL of *o*-Dianisidine buffer solution, 0.3 mL of glucose solution, and 0.1 mL of peroxidase solution were added in sequence. The mixture was shaken evenly with a vortex mixer and preheated in a constant-temperature water bath at 37 °C for 5 min. Then, 0.1 mL of the glucose oxidase solution was added to measure the enzyme activity, and the reaction was carried out accurately at 37 °C for 3 min. Next, 2 mL of 2 mol/L sulfuric acid solution was added to terminate the reaction, and the increase in absorbance at 540 nm was subsequently measured.

The GOD activity in the sample was expressed as X, and the value was expressed as enzyme activity units per gram or enzyme activity units per milliliter (U/g or U/mL). The enzyme activity was calculated using Equation (1):

(1) $$X=\frac{A\times K+C_{0}}{T\times m\times 34.01}\times N$$

where X represents the glucose oxidase activity in the sample, in U/g (or U/mL); A represents the net absorbance of the enzyme reaction group; K represents the slope of the standard curve; C_0_ represents the intercept of the standard curve; N represents the total dilution factor of the sample; M represents the sample weight, in g or mL; T represents the reaction time of 3 min; and 34.01 represents the molecular weight of hydrogen peroxide.

### 2.5. Encapsulation Efficiency

The gel particles were added to the PBS buffer with a pH of 6.0. After being broken down by a homogenizer (5000 rpm, 1 min), the mixture was centrifuged to assess enzyme activity, as referenced by Yang et al. [36]. EE was calculated using Equation (2):

(2) $$EE\;(\%)=\frac{U_{1}}{U_{2}}\times 100$$

where U_2_ is the total GOD activity used for preparing gel particles, and U_1_ is the GOD activity released from gel particles.

### 2.6. Fourier-Transform Infrared (FTIR) Spectroscopy

FTIR analysis of lyophilized gel particles was performed using the KBr pellet method, with the determination wave number ranging from 500 to 3600 cm^−1^, at a scan speed of 2 mm/s and a resolution of 4 cm^−1^ [37]. The data were analyzed using Origin 2019 (Origin Lab, Northampton, MA USA).

### 2.7. Scanning Electron Microscopy (SEM)

The SA/CMC and CS/SA/CMC gel particles were freeze-dried. Under a voltage of 20 kV, the morphology of the gel particles was studied using scanning electron microscopy. The freeze-dried gel particles were fixed on the sample holder with conductive tape adherent. A thin layer of gold was coated under vacuum conditions. The microstructure and morphology were observed at magnifications of 60×, 100×, and 500×. The roughness of the gel particles was analyzed using ImajeJ (Image J, NIH, Bethesda, MD USA) software.

### 2.8. Swelling Capacity

The swelling capacity of gel particles was calculated according to the method of Facin et al. [38]. Firstly, the gel particles were placed in an oven (37 °C, 3 h) for drying. Subsequently, the dried sample was put into a coffee filter paper bag and placed in a PBS buffer with pH values of 2.5 and 7.4 [39]. Incubation was carried out in a shaker at 37 °C and 180 rpm. The gel particles were taken out every 30 min, dried with filter paper to remove water, and weighed. After weighing, the samples were put back into the original PBS solution for continued incubation. The capacity of swelling can be calculated according to Equation (3):

(3) $$Swelling\;capacity=\frac{m_{s}-m_{d}}{m_{s}}\times 100\%$$

where m_s_ refers to the weight of the gel particles after swelling in the buffer, and m_d_ refers to the weight of the gel particles in the dry state.

### 2.9. Thermal Stability

The free enzyme, SA/CMC, and CS/SA/CMC gel particles of glucose oxidase (GOD) were placed in water baths at 60 °C and 70 °C, treated for 10, 20, 30, 40, 50, and 60 min, respectively, and then the enzyme activity was measured. Additionally, the samples were subjected to 80 °C for 2, 4, 6, 8, and 10 min, respectively, followed by enzyme activity measurement. Unheated free enzyme and gel particles served as controls to investigate the thermal stability of the samples. The measurement of enzyme activity was performed as described in Section 2.5.

(4) $$\mathrm{Enzymes}\;\mathrm{heat}\;\mathrm{stability}=\frac{\mathrm{Enzymes}\;\mathrm{residual}\;\mathrm{activity}\;\mathrm{after}\;\mathrm{heating}\;(U/g)}{\mathrm{Enzymes}\;\mathrm{original}\;\mathrm{activity}\;(U/g)}$$

### 2.10. In Vitro Enzyme Release Test

The GOD release test was carried out referencing the method of Ozel et al. [40], with some modifications. The entire experiment process was divided into two stages, including the gastric phase and small intestinal phase. The configuration of simulated gastric fluid (SGF) involved weighing 0.2 g of sodium chloride and 0.32 g of pepsin, which were then placed in a 500 mL beaker. These components were dissolved in distilled water, and the pH was adjusted to 2.5. Finally, the solution was accurately diluted to a final volume of 100 mL in a volumetric flask. The configuration of simulated intestinal fluid (SIF) involved weighing 0.68 g of dipotassium hydrogen phosphate and placing it in a beaker, where it was dissolved in 25 mL of distilled water. Subsequently, 7.7 mL of 0.2 mol/L sodium hydroxide solution, 50 mL of distilled water, and 1 g of trypsin were added to the beaker. The pH value was then accurately adjusted to 6.8 using a pH meter. The prepared gastrointestinal simulated fluid was stored in a refrigerator at 4 °C.

Next, 1 g of gel particles was added to 10 mL of simulated gastric juice and incubated in a shaker at 37 °C and at a rotation speed of 180 rpm. Then, 0.1 mL of the reaction solution was taken out every 30 min for enzyme activity measurement, and 0.1 mL of simulated gastric juice was added after each sampling to maintain the balance of the reaction solution. The entire gastric cycle took 2 h. After the gastric phase was completed, the pH was adjusted to 6.8. The incubation method in the intestinal phase was similar to that in the gastric phase. Each time a sample was collected, an equal volume of simulated intestinal fluid was added. The results were represented by the cumulative release data in the reaction solution. Free GOD served as the control group. The average value of three parallel measurements was calculated for each sample.

### 2.11. Analysis of Storage Stability

The prepared gel particles and free enzymes were placed in a centrifuge tube containing PBS buffer (pH 6.0), and stored at 4 °C. The enzyme activity was determined every 5 days for 30 days. The enzyme activity on the first day was recorded as the maximum activity, and then the relative activity was used to express the enzyme activity.

### 2.12. Statistical Analysis

All data were expressed as means ± standard deviations from three independent experiments. Differences between the means of multiple groups were analyzed via post hoc testing of one-way analysis of variance (ANOVA), alongside Duncan′s multiple range tests. SPSS Statistics 24.0 (IBM, New York, NY, USA) was used to analyze the data.

## 3. Results and Discussion

### 3.1. The EE of GOD Encapsulated in Gel Particles

**Figure 1:**
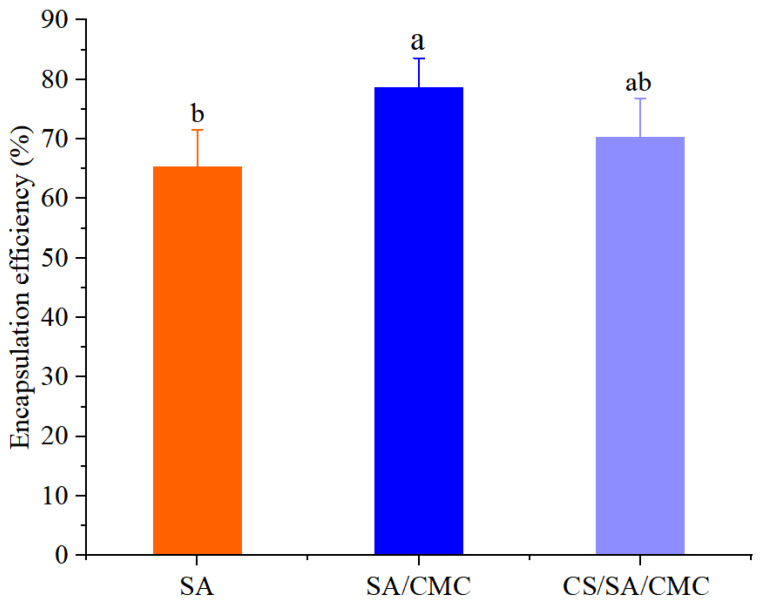
The encapsulation efficiency of glucose oxidase (GOD) encapsulated in gel particles. SA represents SA gel particles loaded with GOD, SA/CMC represents SA/CMC gel particles loaded with GOD, and CS/SA/CMC represents CS/SA/CMC gel particles loaded with GOD. Different letters above the columns indicate that the means of different groups were significantly different (*p* < 0.05) according to one-way analysis of variance.

The EEs of GOD encapsulated in SA gel particles, SA/CMC gel particles, and CS/SA/CMC gel particles are shown in Figure 1.

As shown in Figure 1, the EE of GOD encapsulated in SA gel particles was 65.27%. With the addition of CMC, the EE in SA/CMC gel particles increased to 78.58%, representing an increase of 13.31%. This is because carboxymethyl cellulose possesses excellent water-retaining properties, which reduce water loss during the gel formation process and thereby improve EE. The research conducted by Mai et al. [41] can support our results. The EE of GOD encapsulated in CS/SA/CMC was 70.31%, lower than that of SA/CMC gel particles, suggesting that CS coating was unfavorable for enzyme encapsulation. CS consists of glucosamine units, and the positively charged amino groups in glucosamine interact with the negatively charged carboxyl groups in alginate. This interaction could interfere with the adsorption of GOD onto SA/CMC gel particles, resulting in a reduced EE of GOD [35].

### 3.2. The Gel Particle Structure Analysis According to FTIR

**Figure 2:**
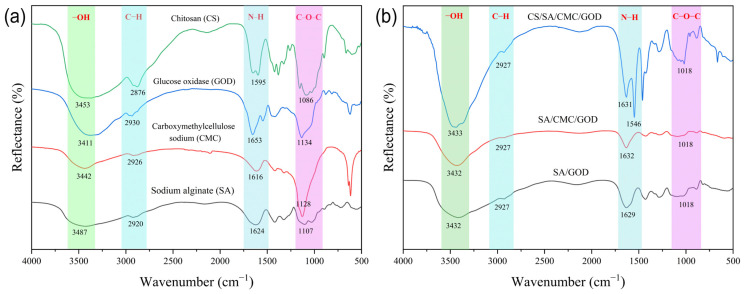
FTIR spectra of the raw biomaterials (**a**) and gel particles (**b**). SA/GOD represents SA gel particles loaded with GOD, SA/CMC/GOD represents SA/CMC gel particles loaded with GOD, and CS/SA/CMC/GOD represents CS/SA/CMC gel particles loaded with GOD.

FTIR can be used to analyze the surface functional groups of biopolymers to determine the intermolecular interactions. The infrared spectra of SA, CMC, SA/GOD, and SA/CMC/GOD are shown in Figure 2.

As shown in Figure 2a, the peaks of the raw biomaterials SA and CMC at 3487 or 3442 cm^−1^, 1107 or 1128 cm^−1^, and 2920 or 2926 cm^−1^ are attributed to the stretching vibrations of -OH, -C-O-C-, and -CH bonds. As shown in Figure 2b, the -OH stretching vibrations of SA (3487 cm^−1^) and CMC (3442 cm^−1^) in SA/CMC gel particles shift to a lower wavenumber (3419 cm^−1^), confirming the presence of intermolecular hydrogen bonding between SA and CMC. This observation is consistent with the findings of Li et al. [42].

For another raw biomaterial (Figure 2a), the characteristic bands of CS were 3411 cm^−1^ (O-H stretching), 2873 cm^−1^ (C-H stretching), and 1595 cm^−1^ (the bending vibration of N-H, representing N-acetylated residues). It is worth noting that, compared to the raw biomaterials, the N-H bending vibration of CS at 1595 cm^−1^ in gel particles shifted to a lower wavelength of 1546 cm^−1^. This shift indicates the formation of electrostatic interactions between CS and SA [43]. The results suggest that chitosan-coated SA/CMC gel particles (CS/SA/CMC) were successfully constructed.

### 3.3. Appearance and Morphology Analysis of Gel Particles

**Figure 3:**
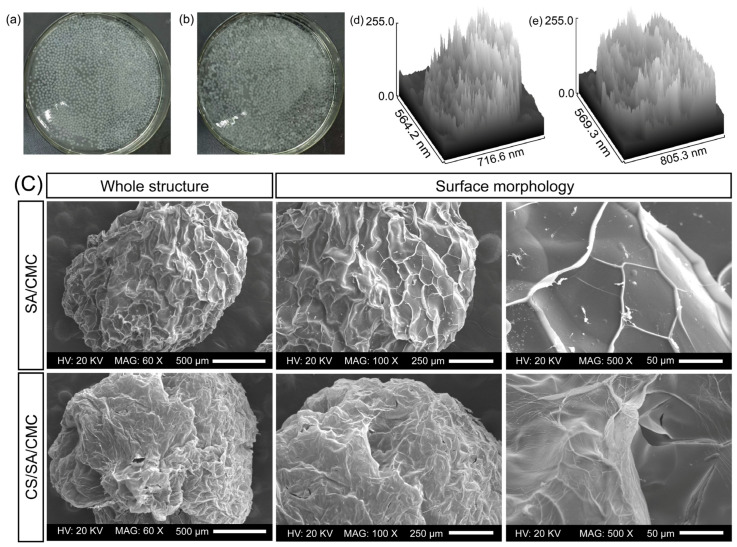
The appearance and morphology of alginate-sodium carboxymethyl cellulose gel particles (SA/CMC) (**a**), SA/CMC gel particles coated with chitosan (CS/SA/CMC) (**b**), and scanning electron microscope (SEM) images (**c**) of the enzyme-loaded gel particles showing a view of the whole structure (60×) and surface morphology (100× and 500×). Surface roughness of SA/CMC gel particles (**d**); surface roughness of CS/SA/CMC gel particles (**e**).

As shown in Figure 3a,b, both SA/CMC gel particles and CS/SA/CMC gel particles were spherical at the millimeter level, with good sphericity and uniform particle size. Among all the gel particles, the main particle size range was concentrated between 0.2 mm and 0.25 mm. After freeze-drying the SA/CMC gel particles and examining by SEM, a comparison with CS/SA/CMC gel particles revealed that the freeze-dried particles predominantly exhibited an oval shape (Figure 3c). Additionally, the surfaces of these particles displayed numerous wrinkles, which is consistent with the findings of Mai et al. [41]. The results indicated that the addition of CMC could lead to the formation of wrinkles on the surface of alginate microparticles, making the microspheres rougher and more wrinkled. Both types of gel particles had unique vein-like patterns on their surfaces, which were formed due to the cross-linking of Ca^2+^ with polysaccharides, which was consistent with the findings of Rao et al. [44]. CS/SA/CMC gel particles possess a denser structure compared to SA/CMC gel particles (Figure 3e), characterized by numerous folds and occasional cracks between these folds. Furthermore, the surface layer exhibits some pores, suggesting that these microspheres can achieve controlled release of the encapsulated active substances [45]. Further research on the surface morphology showed that the venous-like folds on the surface of SA/CMC particles are covered by a thin film, due to the electrostatic interactions between CS and SA/CMC. The benefit of coating SA/CMC gel particles with a layer of chitosan lies in its ability to safeguard GOD from the effects of external physicochemical influences [46].

### 3.4. Swelling Capacity Analysis of Gel Particles

**Figure 4:**
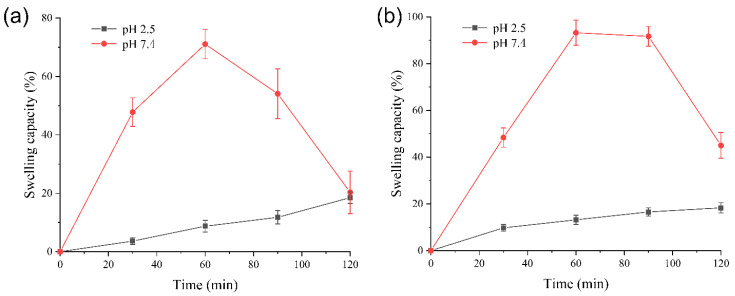
The swelling capacity of dried alginate-sodium carboxymethyl cellulose gel particles (SA/CMC) (**a**) and SA/CMC gel particles coated with chitosan (CS/SA/CMC) (**b**) at different pH values.

Calcium alginate and carboxymethylcellulose are polyelectrolytes, showing pH-dependent swelling. The swelling capacity analysis of gel particles is shown in Figure 4.

As shown in Figure 4, at pH 2.5, the swelling capacities of SA/CMC gel particles and CS/SA/CMC gel particles were relatively small. At 120 min, the swelling capacity was around 18%. However, at a pH of 7.4, as time increases, the two types of gel particles continuously swelled, reaching their maximum swelling at 60 min, with the swelling capacity of 71.06 ± 5.03% and 93.22 ± 5.36%, respectively. After 60 min, the two types of gel particles partially dissolved in the buffer solution at pH 7.5, resulting in a gradual decrease in swelling degree. The two types of gel particles exhibited minimal swelling under acidic conditions, while they demonstrated a significantly higher degree of swelling in neutral or alkaline environments. In neutral or alkaline media, the carboxyl groups in SA and CMC will convert into negatively charged carboxylate ions, causing electrostatic repulsion between different polymer chains, which in turn forces the polymer network to expand [47]. This property facilitated the release of the encapsulated enzyme in the intestine. Three main mechanisms of enzyme release from the alginate matrix include swelling, erosion, and diffusion [48]. Under acidic conditions with a lower pH, the main diffusion mechanisms of the GOD in the GOD-loaded SA/CMC and CS/SA/CMC gel particles could be the chemical-gradient-guided diffusion mechanism and the expansion release mechanism caused by water absorption and the swelling of the alginate matrix [49]. Erosion, another physical mechanism, primarily occurs under neutral or alkaline conditions as the SA/CMC and CS/SA/CMC gel particles gradually wear down, allowing GOD to be released from the gel particles.

### 3.5. Thermal Stability Analysis of GOD

**Figure 5:**
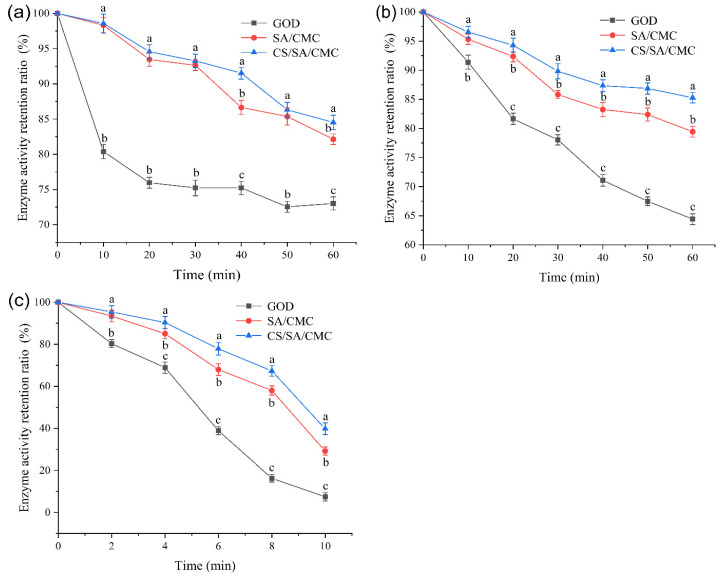
The thermal stability of free glucose oxidase (GOD) and GOD encapsulated in gel particles was evaluated by moist heating at 60 °C for 30 min (**a**), at 70 °C for 30 min (**b**), and at 80 °C for 10 min (**c**). GOD represented the free enzyme of GOD, SA/CMC represented SA/CMC gel particles loaded with GOD, and CS/SA/CMC represented CS/SA/CMC gel particles loaded with GOD. Values with different letters in the same column are significantly different (*p* < 0.05).

The thermal stability of GOD is crucial in the application of animal feed [50]. Enzymes are usually added during the grain processing step, where the feed is subjected to high-temperature extrusion (70 °C drying time < 1.5 h) [51], followed by pelleting. Therefore, we examined the thermal stability of free GOD and encapsulated GOD at 60 °C, 70 °C, and 80 °C. Moisture is crucial in destabilizing enzymes at elevated temperatures because water speeds up the structural changes in proteins by disrupting the intermolecular hydrogen bonds of the enzymes during heat treatment [52]. The enzyme activity retention rates of free GOD and gel particles loaded with GOD at different moist heat temperatures are shown in Figure 5.

As shown in Figure 5, when the GOD was heated at different moist heat temperatures, the enzyme activity retention rates of all encapsulated GOD samples were higher than those of the free enzyme. According to Figure 5a, when the free enzyme was incubated at 60 °C for 30 min, the enzyme activity retention rate was 75.23%. In contrast, when the SA/CMC and CS/SA/CMC gel particles loaded with GOD were incubated at 60 °C for 30 min, the GOD activity retention rates were 92.63% and 93.27%, respectively. Among them, the GOD activity retention rate of the GOD-loaded CS/SA/CMC gel particle was 18.04% higher than that of the free enzyme. As shown in Figure 5b, when the free enzyme and the enzyme-loaded gel particles were incubated at 70 °C for 60 min, the remaining GOD activity of the free enzyme was 64.42%, while the GOD activities of the SA/CMC and CS/SA/CMC gel particles loaded with GOD were still 79.44% and 85.28%, respectively. As shown in Figure 5c, when the free enzyme and the GOD-loaded gel particles were incubated at 80 °C for 10 min, the remaining enzyme activity of the free GOD was 7.5%, while the GOD activities of the SA/CMC and CS/SA/CMC gel particles loaded with GOD were still 29.16% and 39.76%, respectively. The thermal stability of GOD was enhanced by chitosan coating when compared to SA/CMC gel particles; however, this improvement did not positively affect the EE of GOD.

Encapsulating GOD with SA/CMC and CS/SA/CMC gel particles could be an effective approach to improve the thermal stability of GOD. The enhanced thermal stability of the encapsulated GOD was attributed to the structural constraint of the GOD by alginate, which was beneficial for the stabilization of the GOD’s spatial structure under heat treatment conditions, as confirmed in the findings of Weng et al. [53,54]. At the same time, the CS film on the surface of the GOD-loaded CS/SA/CMC gel particles reduced the dissolution of the carrier and the dissociation of the GOD prosthetic group. This enhancement improved the heat resistance of GOD, which aligns with the findings of Wang et al. [55] regarding the immobilization of cellulase using alginate (SA), polyethylene glycol (PEG), and chitosan (CS). A previous study indicated that the thermal stability of phytases improved nearly fourfold (at 90 °C for 1 min) when encapsulated through SA encapsulation [54]. The thermal stability of encapsulated GOD increased by 4.3 times compared to free GOD (at 80 °C for 10 min), indicating that encapsulating GOD in CS/SA/CMC gel particles has certain potential for industrial applications.

### 3.6. The Release Characteristics of GOD Gel Particles Under In Vitro Gastrointestinal Conditions

**Figure 6:**
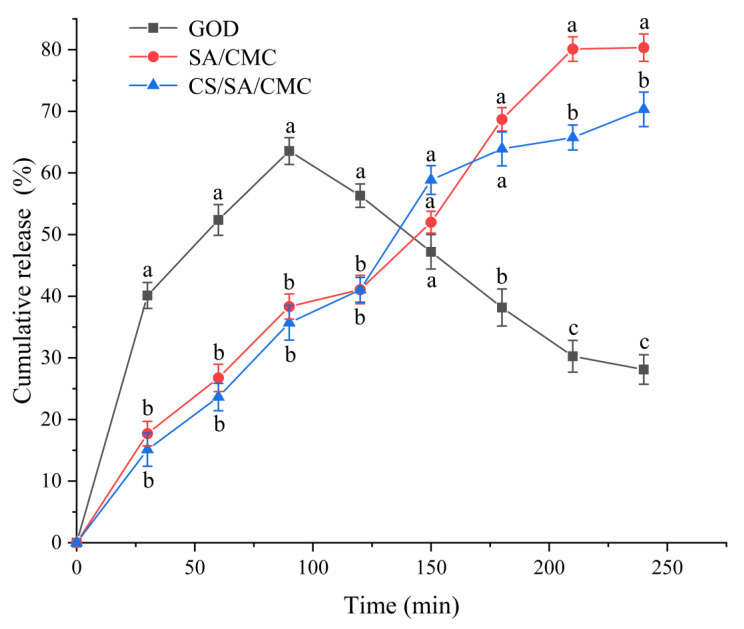
The effect of free glucose oxidase (GOD) and gel particles loaded with GOD in the gastrointestinal tract mimics digestive fluid. GOD represents the free enzyme of GOD, SA/CMC represents SA/CMC gel particles loaded with GOD, and CS/SA/CMC represents CS/SA/CMC gel particles loaded with GOD. Values with different letters in the same column are significantly different (*p* < 0.05).

The biological activity and bioavailability of enzymes are important factors that affect their application. However, in certain cases, the activity and bioavailability of enzymes may be influenced by encapsulation [54]. The study of the biological activity of encapsulated GOD was conducted by releasing GOD in simulated gastrointestinal fluid, which simulated the pH values, physical forces, retention time, and protease conditions of the animal gastrointestinal tract [52]. The cumulative release rate of GOD activity of the prepared gel particles placed in the simulated gastrointestinal fluid is shown in Figure 6.

The results showed that the release rate of free GOD increased rapidly in the first 30 min of the culture medium and reached a maximum of 63.55% after 90 min. Then, it gradually decreased in the small intestine phase, dropping to 28.11% after 240 min. The cumulative enzyme release of SA/CMC gel particles and CS/SA/CMC gel particles loaded with GOD showed a continuous upward trend throughout the gastrointestinal period. The overall trend of enzyme release of CS/SA/CMC gel particles loaded with GOD in the gastric phase was smaller than that of SA/CMC gel particles. CS was adsorbed on the surface of SA through electrostatic deposition, reducing the pores of gel particles [56] and thereby reducing the release of GOD. In the small intestine phase, the GOD could be continuously released. The release amount of enzyme in SA/CMC gel particles loaded with GOD increased from 41.07% to 80.33%, while the release amount of enzyme in CS/SA/CMC gel particles loaded with GOD increased from 41.04% to 70.32%. Similarly, release behavior analysis found that CS/SA gel particles loaded with SOD can effectively release SOD into the simulated intestinal fluid (SIF), and the release rate was faster in the first two hours [56]. The calcium alginate shell was hydrated and swelled in SGF, which greatly promoted the dissolution of gel particles at a slightly alkaline pH [34]. Meanwhile, the initially compact gel network expands and the pores increase in size during the swelling process, allowing for the rapid and extensive release of GOD from gel particles into SIF [57]. This could be the reason for the burst release of enzymes in SIF. In this study, it was found that SA/CMC gel particles could disintegrate in intestinal fluid with a pH of 6.8, whereas CS/SA/CMC gel particles did not disintegrate. The release amount of SA/CMC gel particles loaded with GOD was greater than that of CS/SA/CMC gel particles. The stomach functions as the primary organ for digestion, while the small intestine is chiefly responsible for nutrient absorption. This makes the release of enzymes in the small intestine particularly crucial [58]. These results indicate that SA/CMC and CS/SA/CMC gel particles offer physical protection, effectively preventing the degradation of GOD by proteases. Additionally, the encapsulation of GOD using SA/CMC and CS/SA/CMC gel particles does not negatively impact the enzyme’s bioavailability.

### 3.7. Storage Stability Analysis of GOD Gel Particles

**Figure 7:**
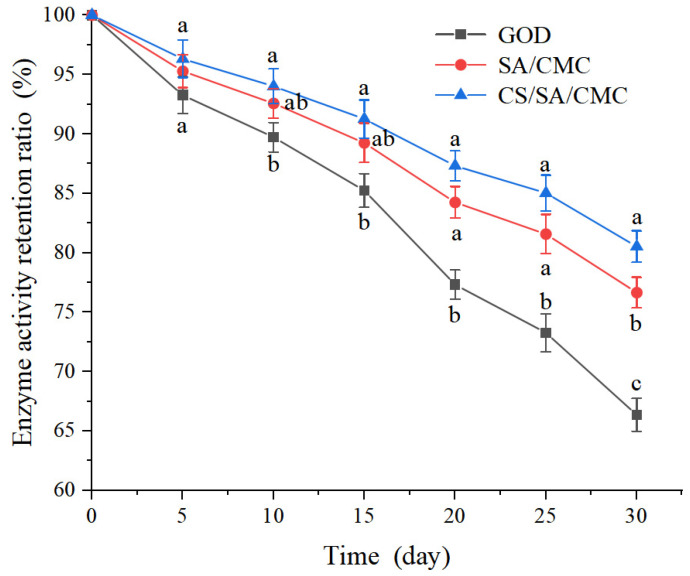
Storage stability of free glucose oxidase (GOD) and gel particles loaded with GOD at 4 °C for 30 days. GOD represents the free enzyme of GOD, SA/CMC represents SA/CMC gel particles loaded with GOD, and CS/SA/CMC represents CS/SA/CMC gel particles loaded with GOD. Values with different letters in the same column are significantly different (*p* < 0.05).

Effectively prolonging the storage time of GOD can not only reduce the waste of resources but also contribute to ensuring the stable supply of industrial production. Therefore, the length of storage time is of great significance to the recovery rate of the enzyme. The retention rates of enzyme activity of free enzymes and gel particles of GOD at different storage times are shown in Figure 7.

The result showed that with the extension of the placement time, the activity of the enzyme gradually decreased. After 30 days, the activity of the free GOD dropped to 66.35%. Encapsulation could significantly improve the storage stability of the enzyme after 30 days. The enzyme activities of SA/CMC gel particles and CS/SA/CMC gel particles loaded with GOD were 76.65% and 80.52%, respectively. This enhancement could be due to the combination with the carrier preventing the possible distortion effect of the buffer solution on the active site of the enzyme [59]. After 30 days, CS/SA/CMC gel particles loaded with GOD showed higher enzyme activity than SA/CMC gel particles. The enhancement could be attributed to the interaction between protonated amines in CS with SA and CMC. The interaction forms an electrolyte-film-coating layer on the surface of the gel particles [60] and reduces their porosity [61,62], thereby enhancing the protective effect of the GOD under storage conditions. The results indicate that encapsulating GOD in SA/CMC and CS/SA/CMC gel particles could enhance the storage stability of GOD, showing potential applications in commercial water-based products such as fish feed.

## 4. Conclusions

In this study, the synergy between SA and CMC was used to encapsulate GOD to enhance the encapsulation efficiency of GOD. The addition of CMC can improve the EE of GOD in alginate gel particles. On this basis, CS was added to coat the SA/CMC gel particles to further improve the stability of GOD. GOD’s encapsulation in alginate gel particles (SA/CMC and CS/SA/CMC) was proven to improve its thermal and storage stability. Furthermore, the favorable bioactivity of the encapsulated GOD was achieved through controlled enzyme release, suggesting that the developed encapsulation method could have significant potential for application in animal feed, particularly in fish feed. In addition, SA/CMC and CS/SA/CMC gel particles have the potential to encapsulate active food ingredients and other bioactive compounds as functional supplements.

## Data Availability

The original contributions presented in the study are included in the article, further inquiries can be directed to the corresponding author.

## Supplementary Materials

The following supporting information can be downloaded at: https://www.mdpi.com/article/10.3390/foods14040664/s1, Figure S1: Single-factor experiment on the encapsulation of glucose oxidase (GOD) in sodium alginate and carboxymethyl cellulose (SA/CMC) gel particles; the effect of sodium alginate concentration on the encapsulation effect of GOD (a); the effect of sodium carboxymethyl cellulose concentration on the encapsulation effect of GOD (b); the effect of GOD concentration on the encapsulation effect of GOD (c); the effect of encapsulation time on the encapsulation effect of GOD (d); the effect of CaCl2 concentration on the encapsulation effect of GOD (e), Figure S2: Single-factor experiment on chitosan (CS)-coated sodium alginate and carboxymethyl cellulose (CS/SA/CMC) gel particles encapsulating glucose oxidase (GOD); the effect of CS solution concentration on the encapsulation effect of GOD (a); the effect of CS solution pH on the encapsulation effect of GOD (b); the effect of coating time on the encapsulation effect of GOD (c), Figure S3: Three-dimensional response surfaces and the contour plot for the effects of the chitosan solution concentration, chitosan solution pH and coating time on the encapsulation efficiency; Three-dimensional response surfaces and the contour plot for the effects of the chitosan solution concentration, chitosan solution pH on the encapsulation efficiency (a,b); Three-dimensional response surfaces and the contour plot for the effects of the chitosan solution concentration and coating time (c,d); Three-dimensional response surfaces and the contour plot for the effects of chitosan solution pH and coating time (e,f), Table S1: Level table of orthogonal experimental factors, Table S2: The results of orthogonal experiment, Table S3: Factors and coded levels of Box-Behnken design, Table S4: Arrangement and result of Box-Behnken design, Table S5: Regression model analysis of variance using the encapsulation efficiency of glucose oxidase as response value, Table S6: Estimated regression coefficients for the quadratic polynomial model.
