# Supplementary material for: Enhanced Thermal and Storage Stability of Glucose Oxidase via Encapsulation in Chitosan-Coated Alginate and Carboxymethyl Cellulose Gel Particles
Source: Foods. 2025 Feb 15;14(4):664. doi: 10.3390/foods14040664 (PMC11854346; doi:10.3390/foods14040664)
Supplement: Supplementary file 1 [file foods-14-00664-s001.zip › foods-3369306-supplementary.pdf]

Foods

Encapsulation of glucose oxidase (GOD) in chitosan-coated gel particles of alginate and carboxymethyl cellulose for enhanced thermal and storage stability

Zhihao Guo, Jian Ren, Chunli Song *

1 College of Food and Bioengineering, Qiqihar University, Qiqihar, China

2 Engineering Research Center of Plant Food Processing Technology, Ministry of Education, Qiqihar, China

*Correspondence author:

Prof. Chunli Song, College of food and bioengineering, Qiqihar University, Qiqihar, Heilongjiang Province, China

E-Mail: songchunli@qqhru.edu.cn (Chunli Song)

1 Supplementary Table

Table S1 Level table of orthogonal experimental factors

Level	Factors			
	A Sodium alginate concentration (%)	B Sodium carboxymethyl cellulose concentration (%)	C $CaCl_2$ concentration (%)	D Encapsulation time (min)
1	1.5	0.05	0.5	15
2	2	0.1	1	30
3	2.5	0.15	1.5	45

Table S2 The results of orthogonal experiment

Experiment number	Factors				Encapsulation efficiency (%)
	A	B	C	D	
1	1	1	1	1	92.23
2	1	2	2	2	78.58
3	1	3	3	3	72.52
4	2	1	2	3	61.68
5	2	2	3	1	77.39
6	2	3	1	2	74.31
7	3	1	3	2	72.80
8	3	2	1	3	67.95
9	3	3	2	1	64.80
K_1	81.110	75.570	78.163	78.140	
K_2	71.127	74.640	68.353	75.230	
K_3	68.517	70.543	74.237	67.383	
R	12.593	5.027	9.810	10.757	
Optimal level	A_1	B_1	C_1	D_1	
Factors affecting the level			A>D>C>B		

Table S3 Factors and coded levels of Box-Behnken design

Factors	Coding and Level		
	-1	0	1
A Chitosan solution concentration (%)	0.4	0.6	0.8
B Chitosan solution pH	3.5	4	4.5
C Coating time (min)	20	30	40

Table S4 Arrangement and result of Box-Behnken design

Experiment number	A	B	C	Encapsulation efficiency (%)
1	0	-1	1	56.14
2	0	0	0	73.04
3	-1	0	1	58.79
4	-1	-1	0	50.28
5	-1	0	-1	54.78
6	1	0	-1	40.92
7	0	1	1	35.97
8	0	0	0	72.64
9	0	0	0	69.99
10	0	0	0	68.39
11	0	0	0	67.49
12	0	-1	-1	53.73
13	1	0	1	50.43
14	0	1	-1	52.88
15	-1	1	0	47.81
16	1	1	0	36.60
17	1	-1	0	50.03

Table S5 Regression model analysis of variance using the encapsulation efficiency of glucose oxidase as response value

Source	Sum of Squares	df	Mean Square	F-value	P-value
Model	2095.00	9	232.78	11.60	0.0019
A	141.79	1	141.79	7.06	0.0326
B	170.39	1	170.39	8.49	0.0225
C	0.1200	1	0.1200	0.0060	0.9405
AB	30.03	1	30.03	1.50	0.2609
AC	7.56	1	7.56	0.3767	0.5588
BC	93.32	1	93.32	4.65	0.0680
A^2	536.69	1	536.69	26.74	0.0013
B^2	694.17	1	694.17	34.58	0.0006
C^2	255.51	1	255.51	12.73	0.0091
Pesidual	140.51	7	20.07		
Lack of Fit	115.89	3	38.63	6.28	0.0541
Pure Error	24.62	4	6.16		
Cor Toal	2235.51	16			

Table S6 Estimated regression coefficients for the quadratic polynomial model

Project	Std.Dev	Mean	C.V.%	
Numerical value	4.48	70.63	6.34	
Project	R^2	Adjusted R^2	Predicted R^2	Adeq Precision
Numerical value	0.9371	0.8563	0.1533	10.3878

2 Supplementary Figure

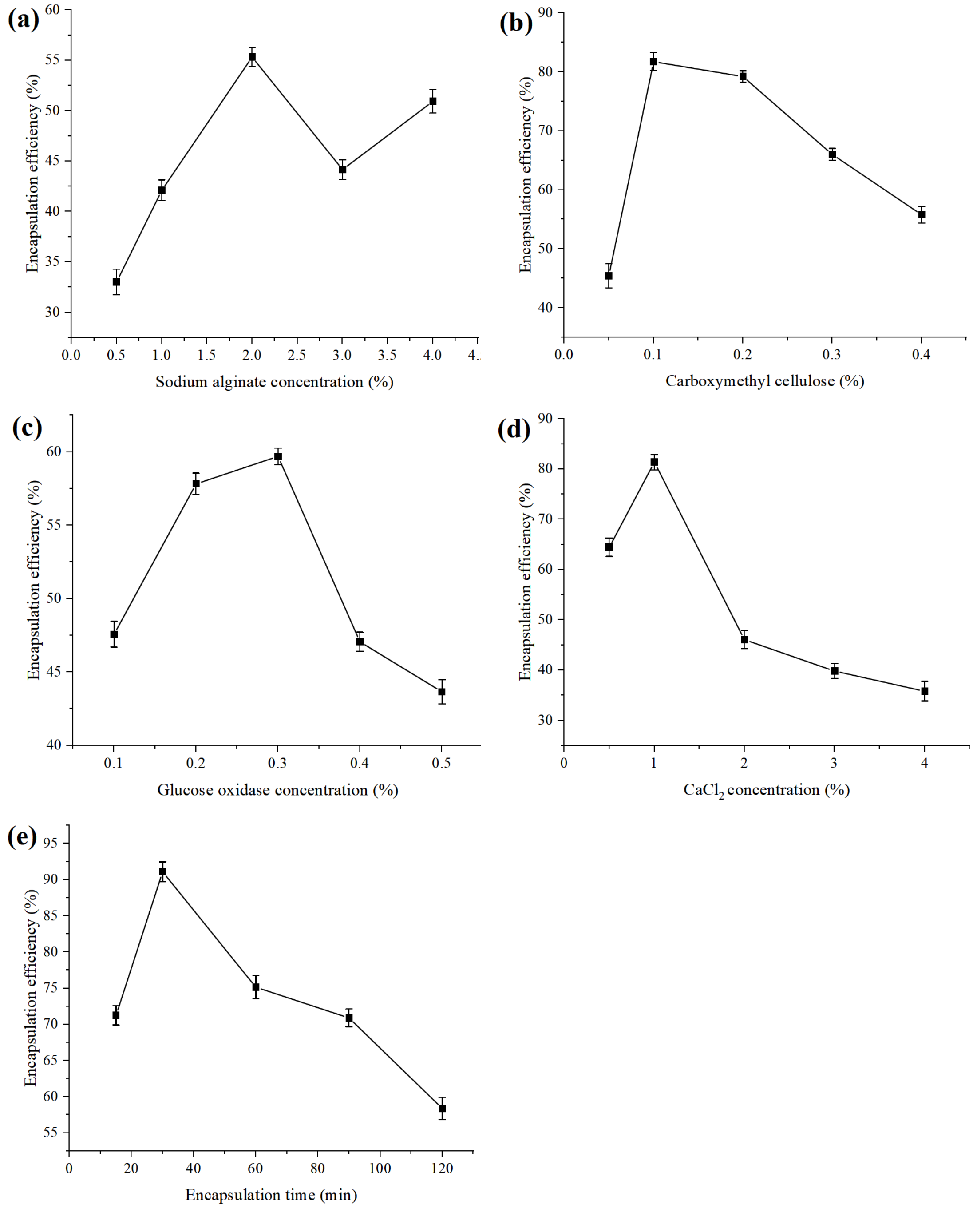

Figure S1. Single-factor experiment on the encapsulation of glucose oxidase (GOD) in sodium alginate and carboxymethyl cellulose (SA/CMC) gel particles; the effect of sodium alginate concentration on the encapsulation effect of GOD (a); the effect of sodium carboxymethyl cellulose concentration on the encapsulation effect of GOD (b); the effect of GOD concentration on the encapsulation effect of GOD (c); the effect of encapsulation time on the encapsulation effect of GOD (d); the effect of $CaCl_2$ concentration on the encapsulation effect of GOD (e).

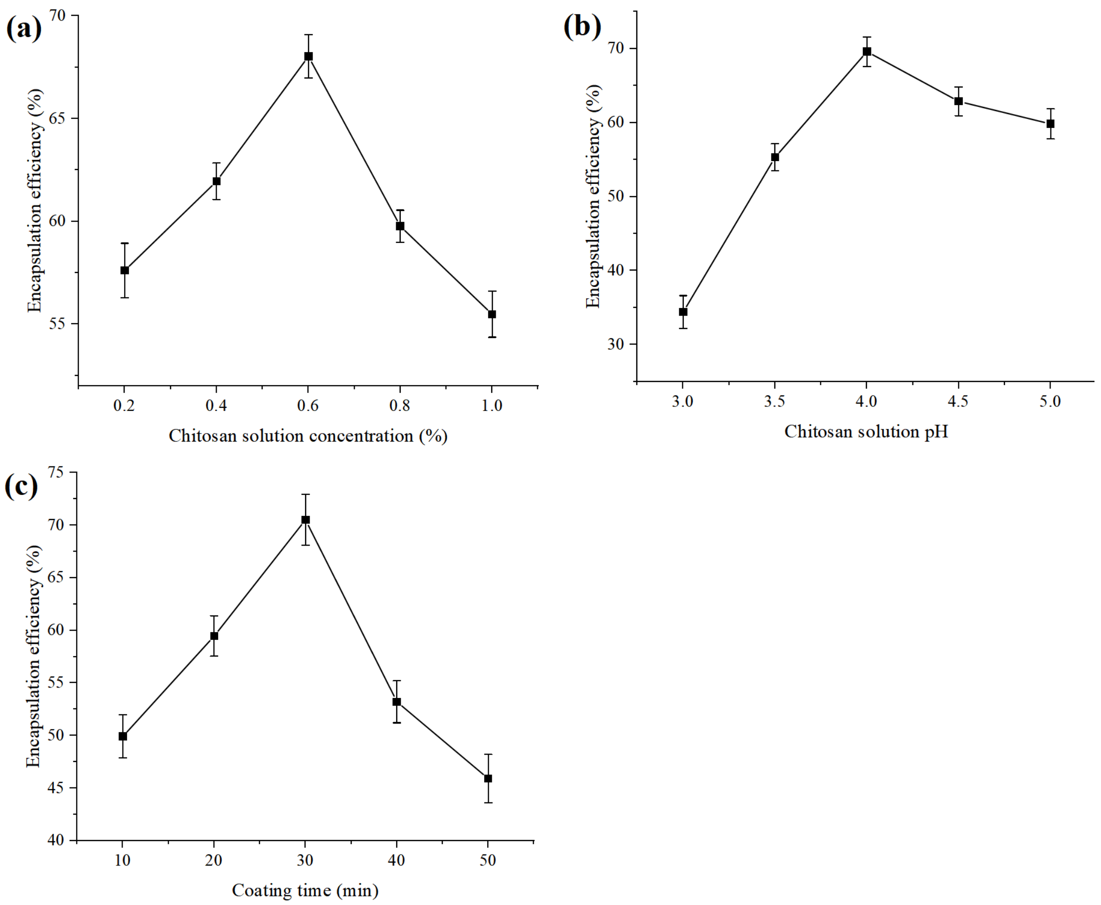

Figure S2. Single-factor experiment on chitosan (CS)-coated sodium alginate and carboxymethyl cellulose (CS/SA/CMC) gel particles encapsulating glucose oxidase (GOD); the effect of CS solution concentration on the encapsulation effect of GOD (a); the effect of CS solution pH on the encapsulation effect of GOD (b); the effect of coating time on the encapsulation effect of GOD (c).

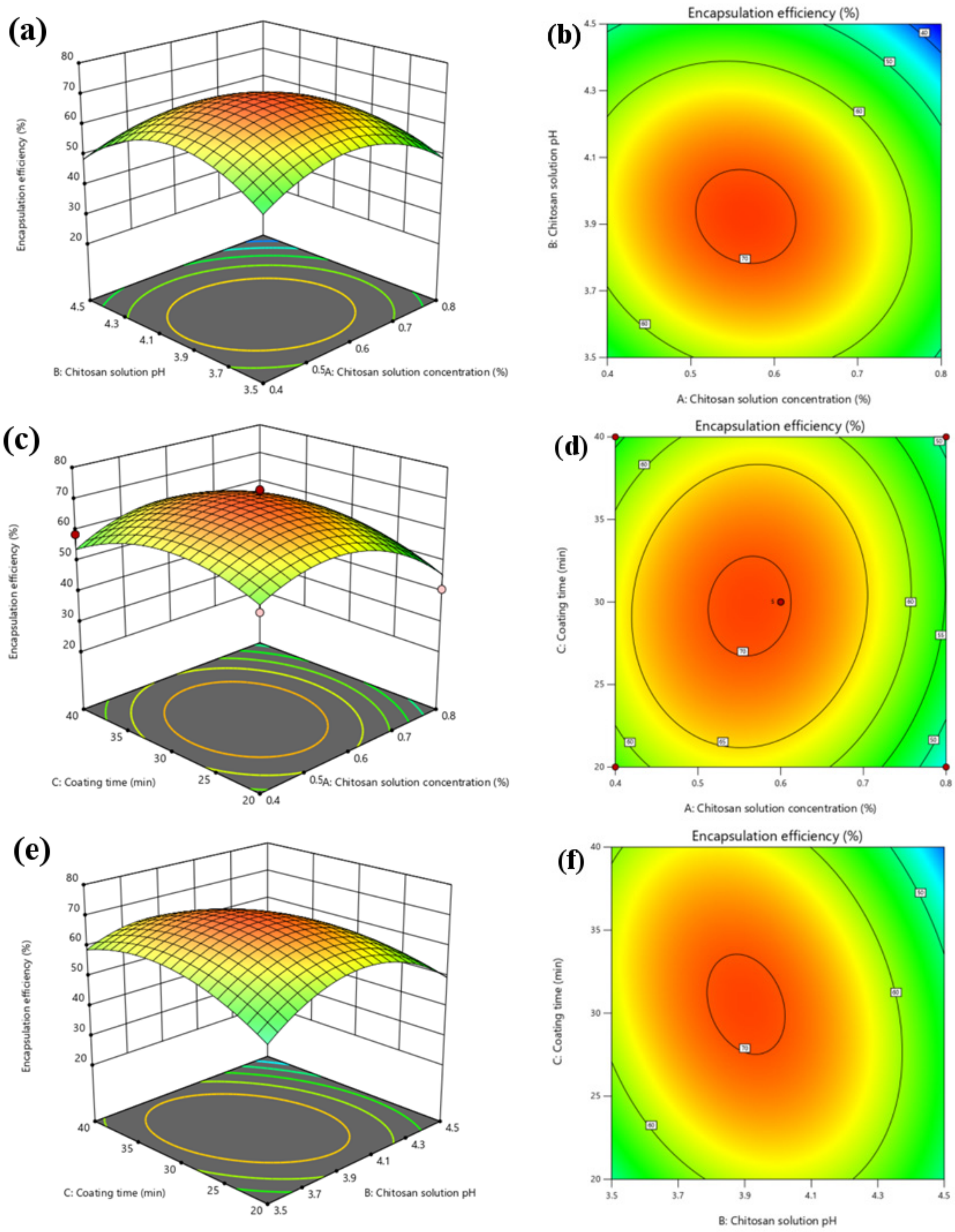

Figure S3. Three-dimensional response surfaces and the contour plot for the effects of the chitosan solution concentration, chitosan solution pH and coating time on the encapsulation efficiency; Three-dimensional response surfaces and the contour plot for the effects of the chitosan solution concentration, chitosan solution pH on the encapsulation efficiency (a) (b); Three-dimensional response surfaces and the contour plot for the effects of the chitosan solution concentration and coating time (c) (d); Three-dimensional response surfaces and the contour plot for the effects of chitosan solution pH and coating time (e) (f).
